# The Gaze Communications Between Dogs/Cats and Humans: Recent Research Review and Future Directions

**DOI:** 10.3389/fpsyg.2020.613512

**Published:** 2020-12-18

**Authors:** Hikari Koyasu, Takefumi Kikusui, Saho Takagi, Miho Nagasawa

**Affiliations:** ^1^Laboratory of Human-Animal Interaction and Reciprocity, Azabu University, Kanagawa, Japan; ^2^Japan Society for the Promotion of Science, Tokyo, Japan

**Keywords:** dogs, cats, humans, gaze, interaction, communication, bond

## Abstract

Dogs (*Canis familiaris*) and cats (*Felis silvestris catus*) have been domesticated through different processes. Dogs were the first domesticated animals, cooperating with humans by hunting and guarding. In contrast, cats were domesticated as predators of rodents and lived near human habitations when humans began to settle and farm. Although the domestication of dogs followed a different path from that of cats, and they have ancestors of a different nature, both have been broadly integrated into—and profoundly impacted—human society. The coexistence between dogs/cats and humans is based on non-verbal communication. This review focuses on “gaze,” which is an important signal for humans and describes the communicative function of dogs’ and cats’ eye-gaze behavior with humans. We discuss how the function of the gaze goes beyond communication to mutual emotional connection, namely “bond” formation. Finally, we present a research approach to multimodal interactions between dogs/cats and humans that participate in communication and bond formation.

## Domestication of Dogs and Cats

Dogs (*Canis familiaris*) and cats (*Felis silvestris catus*) are the closest animals living with humans. Dogs, domesticated approximately 15,000 years ago, were the first animals domesticated from wild species (Freedman and Wayne, 2017). They acquired social tolerance to humans and cooperated with humans by assisting in hunting and guarding. Dogs were first selected because of their reduced stress response to humans, and then for the usefulness of their cooperation with humans (Driscoll et al., 2009). Wolves (*Canis lupus*), sharing a common ancestor with dogs, have developed a greater ability to cooperate than dogs, but wolves only display intra-specific cooperation. In contrast, cats were domesticated approximately 10,000 years ago, primarily because they were predators whose prey included rodents (Vigne et al., 2004). Additionally, cats were not artificially selectively bred. The cat’s ancestor is the wildcat (*Felis silvestris lybica*), a solitary, territorial animal, like most other Felidae (Bradshaw, 2016).

**FIGURE 1:**
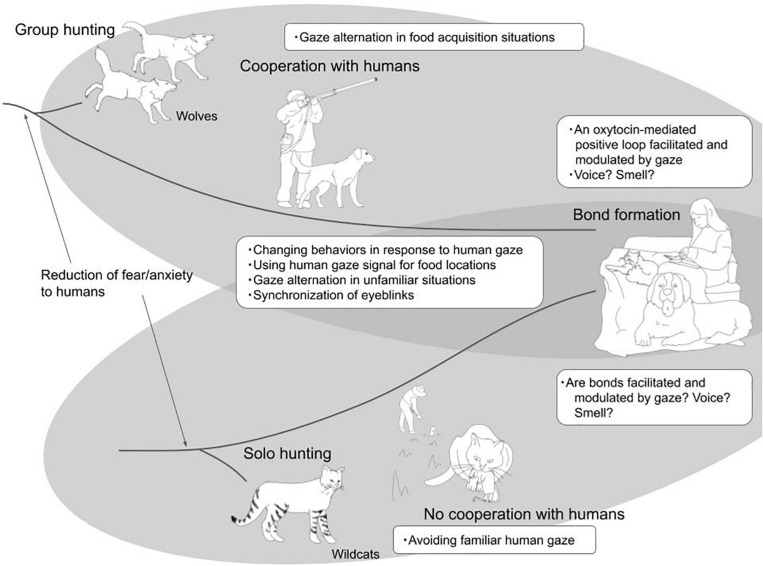
Domestication of dogs and cats and gaze communication with humans. Although dogs and cats have ancestors with a different nature, they have started living with humans, using nonverbal communication, especially gaze. The similar gaze communications observed in both dogs and cats were shown in the overlap of two circles. Using non-verbal communicative signals they can form bonds with humans.

Although dogs have ancestors with a different nature than cats, and were domesticated via different processes, they are the most common animals living with humans (Figure 1). The foundation of this coexistence is non-verbal communication. They use their sense of smell, hearing, touch, and eyesight to communicate with humans. Dogs and cats are sensitive to gaze, which humans use as a form of non-verbal communication. In this review, we introduce the communicative function of gaze in dog-human and cat-human interactions in recent studies (Table 1). Second, we describe bond formation beyond communication and the importance of gaze in bond formation. Finally, we discuss the possibility that other senses contributed to the bonds formed between dogs/cats and humans and a research approach to the multimodal interactions that facilitate communication and bond formation. Comparing the ways dogs and cats interact with humans provides insight into how both species have integrated into human society. In other words, the differences between dogs and cats may have originated due to differences in their ancestral species’ social natures and the process of domestication. The similarities between dogs and cats may also be due to changes in their cognitive function that allowed them to integrate into human society. With these considerations in mind, we review the findings to date.

**TABLE 1:** Gaze communication between dogs/cats and humans.

Dogs	References	Cats	References
**Response to human gaze**			
Stole food less often	Call et al., 2003; Kaminski et al., 2013	Avoided the gaze of familiar human	Koyasu and Nagasawa, 2019
Obeyed more commands of their owners	Schwab and Huber, 2006	Selected food from humans who looked at them	Ito et al., 2016
Fetched the toy that humans could see in the situation with two toys	Call et al., 2003		
Increased attention-getting behaviors	Ohkita et al., 2016		
Selected food from humans who looked at them	Gácsi et al., 2004		
**Using human signals**			
Used human pointing in the task of selecting one of two containers	Miklosi et al., 2005	Used human pointing in the task of selecting one of two containers	Miklosi et al., 2005
Used human gaze direction with pointing in the task of selecting one of two containers	Hare et al., 2002	Looked in the direction indicated by human gaze (with head movements)	Pongrácz et al., 2019
Looked in the direction directed by human gaze (with head movements)	Hare et al., 1998; Agnetta et al., 2000; Téglás et al., 2012; Met et al., 2014	Followed the container that humans visited in a situation with two food containers	Chijiiwa et al., 2020
Followed the container that humans visited in a situation with two food containers	Chijiiwa et al., 2020; Nagasawa et al., 2020		
**Social reference**			
Looked alternately at the food and the owner when it could access the food	Miklosi et al., 2005, Lazzaroni et al., 2020	Did not look alternately at the food and the owner when it could not access the food	Miklosi et al., 2005
Looked alternately at the strange object and the owner	Merola et al., 2012a,b	Looked alternately at the strange object and the owner	Merola et al., 2015
**The role of gaze in bond formation**			
Increased attention-getting behaviors in dogs, which function as attachment behaviors in response to human gaze	Ohkita et al., 2016	Eyeblink synchronization during mutual gazing	Koyasu et al., 2020
Dog owner’s oxytocin secretion increased in response to the dog’s gaze	Nagasawa et al., 2009		
An oxytocin-mediated positive loop of bond formation facilitated and modulated by gazing, like mother-infant	Nagasawa et al., 2015		
Eyeblink synchronization during mutual gazing	Koyasu et al., 2020		

## Gaze Communication for Dogs

It is easy to determine the direction of gaze of wolves that hunt cooperatively in packs because of the type of eyes they have. Furthermore, wolves have developed behaviors for receiving and sending their gaze signals (Ueda et al., 2014). These results suggest that wolves use gaze to communicate with others. It is possible that dog’s ancestors inherited this function and dogs have applied it as gaze-based communication in their interactions with humans.

When dogs receive human gaze, they change their behavior depending on its direction. For example, dogs were given a series of trials in which they were forbidden to consume any visible food (Call et al., 2003; Kaminski et al., 2013). When the human looked at them, dogs retrieved food less often than in the conditions in which humans did not look at them. Additionally, dogs were more obedient to their owners’ commands when their owners looked at them (Schwab and Huber, 2006). Dogs detect a human’s attentional state from the direction of their gaze. This behavior is necessary for dogs’ ability to respond to human commands, such as when hunting. Furthermore, dogs expect what humans can see and change their behavior accordingly. For example, in an experiment, two toys were placed in a room, one was blocked by opaque panels to prevent humans from seeing it. Although the dogs could see both toys, when ordered to “fetch,” they picked the one visible to the humans (Call et al., 2003). In another experiment observing the free behaviors of dogs, the duration of their attention-getting behaviors (e.g., whining, whimpering, and looking at owners’ faces) was longer in response to their owner’s gaze (Ohkita et al., 2016). This ability to recognize other’s perspectives could be beneficial to humans’ and dogs’ cooperative hunting.

Dogs are more likely to select food from humans who are looking at them than those who are not (Gácsi et al., 2004); this tendency was likely to have been acquired early in the domestication process. Dogs often exhibit more hesitative behaviors when approaching a blindfolded human but beg for food from a human with visible eyes (Gácsi et al., 2004). This difference indicates that, based on gaze, dogs may distinguish between humans who are willing to give food and those who are not, and they receive food from the former when they are allowed to consume. Food, and knowing if someone would give them food, was a significant factor in dogs’ early domestication.

Dogs use human signals such as pointing (Miklosi et al., 2005) and gazing accompanied by pointing even when they are puppies (Hare et al., 2002). One study showed that dogs were easily able to use human pointing to select one of two containers in which food was hidden using human pointing and gaze direction. In contrast, chimpanzees, close relatives of humans, find it difficult to use human cues in this task. Furthermore, a dog’s gaze follows only a human gaze (Hare et al., 1998; Agnetta et al., 2000; Téglás et al., 2012; Met et al., 2014). They look in the direction in which the human gaze (with head movements) is directed. Dogs follow not only the gaze but also the movements of humans. When there were two bowls with food, dogs followed the one that more humans went to under certain conditions (Nagasawa et al., 2020). Dogs chose the same container they had seen humans choose, even if they had seen the human removing food from it and pretending to eat it (Chijiiwa et al., 2020). Following human behaviors, including gaze, would have helped dogs-human cooperation for hunting and gathering before humans began establishing cultures based on cultivation and settlements.

Dogs exhibit social referencing by looking at and using facial expressions and behaviors of others in unfamiliar situations. In unsolvable tasks in which they cannot access food, they look at their owners (Miklosi et al., 2005), but see Lazzaroni et al. (2020). Even when they encounter a strange object, a fan with some ribbons, most dogs look referentially to their owners after looking at the strange object (Merola et al., 2012a,b). This alternating gaze is thought to have the function of joint attention, directing others’ gaze to an object to garner problem-solving cooperation. In humans, alternating gaze followed by joint attention is thought to be related to identifying intention and establishing reference (Emery, 2000). Dogs can also use their own gaze to guide a human’s gaze. Although the function of dogs’ alternating gaze from objects to humans is unclear, dogs may rely on humans to help them in situations where they did not know what to do or cannot solve problems themselves.

Recent research suggests that dogs’ gazing behaviors with humans are influenced by the dogs’ life experiences (Marshall-Pescini et al., 2017; Brubaker et al., 2019). Hence, both domestication and socialization influence a dogs’ gaze behavior with humans.

## Gaze Communication for Cats

The ancestors of cats lived alone; therefore, they may not have needed the ability to read the gaze of other individuals as much as species that hunted in groups. However, there are some recent reports of communication through gaze between cats and humans.

Cats detect human gaze with head movements and accordingly change their behavior (Koyasu and Nagasawa, 2019). When a familiar human (i.e., experimenter) and a cat spent time in the same room, the cat’s behavior was observed in response to the familiar human’s gaze. Cats looked at a familiar human for a shorter duration when the cats were directed gaze than when the cats were not, suggesting that, unlike dogs, they exhibit the behavior of avoiding a familiar gaze. Cats may see a human gaze as the same thing as a cat’s gaze, which indicates a threat in a social situation with no goal or threat (Bradshaw, 2016).

However, in a study with feeding situations, cats were fed by humans who gazed at them (Ito et al., 2016). As with Gácsi et al. (2004), two humans performed differently in front of cats. Cats selected more food from humans who called their names with gazing than food from humans who called their names without gazing. Whether or not cats avoid/select gaze may depend on the experimental situation. Cats also use human signals (Miklosi et al., 2005). Regarding the ability to use human pointing, no statistically significant performance has been found between dogs and cats.

Furthermore, cats can follow a human’s gaze (Pongrácz et al., 2019). In a two-way food selection situation, cats followed a human gaze (with a head movement) in about 70% of the trials. In the condition for selecting one of two food bowls, cats also followed the movements of humans, not just their gaze. As with dogs, cats visited the container following humans, even after seeing the human removing food and pretending to eat it (Chijiiwa et al., 2020). As cats became a part of human society to catch mice but were not required to serve any other role, they may have acquired these abilities in their development because of their dependence on humans for food today. These similarities between dogs and cats indicate they are both easily affected by human behaviors in situations involving food, despite the two species’ different domestication histories.

Cats did not exhibit social reference behavior in the unsolvable task in a feeding situation, unlike dogs (Miklosi et al., 2005). Cats may use the cues provided but not demand cues themselves. Because they did not rely on others for food, they do not demand cues themselves. However, depending on the situation, cats do exhibit social reference (Merola et al., 2015). When cats were shown the fan with some ribbons, 80% alternated their gaze between the fan and their owner, but their behaviors changed based on the human’s emotional expression. When cats encounter strange objects and do not know what to do, they can read the human’s facial expression/behavior or lead the human gaze to objects. Considering the process of domestication, these results may be due to cats’ lack of a history of cooperating with humans to acquire food.

Thus, cats avoid/select the gaze and exhibit/do not exhibit social references depending on the social context. Investigating the contexts in which they require a human gaze may clarify the factors that facilitate the acquisition of human-like communication skills, but cats undoubtedly use gaze to communicate with humans. It is considered to have evolved through life with humans, although the gaze function originally was not necessary for cats.

## Bond Formation Between Dogs/Cats and Humans

Dogs/cats can distinguish between signals based on human emotions. Dogs change their behavior depending on the emotional state of humans. In one study, dogs sniffed, nuzzled, and licked a human who was pretending to cry (Custance and Mayer, 2012). In the social referencing experiment described above, dogs approached the strange fan when their owners reacted positively and moved away from it when their owners reacted negatively (Merola et al., 2012b). Dogs also distinguished between emotional states and facial expressions of humans (Nagasawa et al., 2011; Buttelmann and Tomasello, 2013; Turcsán et al., 2015). Likewise, cats change their behavior depending on the human emotional state. When an owner was depressed, the cat rubbed against their owner more often (Rieger and Turner, 1999). A study showed that a cat’s behavior toward its owner during interactions was affected by their owner’s emotional state (Turner and Rieger, 2001). As with dogs, they distinguished between humans’ facial expressions and associated postures (Merola et al., 2015; Galvan and Vonk, 2016) and strangers’ voices (Quaranta et al., 2020). Dogs/cats can distinguish between signals based on human emotions, which would be the foundation for forming emotional bonds.

Previous studies also suggest that emotional bonds exist between dogs/cats and humans. To examine whether an emotional bond is formed, it is necessary to know if animals show 1) an emotional or behavioral response to specific individuals and 2) a stress response to separation and a stress reduction/pleasurable behavior in reunions (DeVries, 2002). The Ainsworth Strange Situation Test (SST) has been widely used to demonstrate infants’, dogs’, and cats’ bonds to their primary caretakers.

In novel environments, dogs’ exploring and playing behaviors increased when there was an owner in the room compared to when there was only a stranger in the room, and their following behavior increased when the owner left the room compared to when the stranger left the room (Topal et al., 1998). This observation means that dogs behave differently toward their owners than strangers; their owners function as a secure base, like the human mother-infant bond.

In the formation of these human-dog bonds, gaze plays an important role. In experiments observing the free behaviors of dogs in response to human gaze, dogs’ attention-gaining behaviors increased when owners looked at them (Hare et al., 2002). Dogs’ increased attention-seeking when receiving a human gaze may be an attachment signal to draw their owners to them. Furthermore, the dogs’ gaze directed at their owners led to increased oxytocin secretion in their owners (Nagasawa et al., 2009). The oxytocin neuroendocrine system is associated with uterine contraction during childbirth and the promotion of breast milk secretion and plays an important role in maternal behaviors following birth (Nagasawa et al., 2012). A dog’s gaze increased the owner’s interaction with the dog, which increased oxytocin secretion in dogs; in other words, there is an oxytocin-mediated positive loop of bond formation facilitated and modulated by gazing in human-dogs, like mother-infants (Nagasawa et al., 2015).

Cat-owner bonds are a form of attachment similar to that between dogs or infants and their caretakers (Edwards et al., 2007). In the SST, cats have been shown to spend more time engaged in locomotion/exploration when accompanied by their owners and exhibited higher alert behavior event frequency when accompanied by strangers. In a study reexamining these bonds using a crossover design experiment with an improved and counterbalanced modification of the SST, cats vocalized more when owners left the room than when strangers left (but there was no other evidence of a secure base) (Potter and Mills, 2015). Recently, a secure base test (SBT) was conducted to investigate whether humans function as an attachment figure for cats (Nagasawa et al., 2009). The cat-human bond was found to be similar to mother-infant and dog-human bonds; however, additional experimentation with strangers is required due to the lack of evidence that the bond was to a specific individual. There was proximity seeking, separation distress, and reunion behavior, which are indicators of attachment relationships between cats and caretakers. As shown, there is some evidence that there are cat-human bonds. However, it is unclear whether gaze facilitates the bond formation, as in a dog-human relationship. Since cats also communicate through gaze, especially with humans, gaze may be an important factor in bond formation.

Eyeblink, an unconscious signal, may also play a role in the mutual gaze that facilitates bond formation. A study reported that during mutual gaze, eyeblinks were synchronized between dogs and humans (Koyasu et al., 2020). Dogs blinked about one second after their owner or a stranger blinked. The owners blinked immediately after the dogs had blinked, and the strangers blinked after some delay following the dogs’ blinks. Although there was some time lag, the presence of mutual blink synchronization was suggested. The same phenomenon was observed in cats. This synchronization is considered to lead to a mutual understanding and effective communication in humans. Synchronizing and obtaining the same physiological state as others may also lead to mutual understanding and effective communication in dogs and cats.

These results suggest that a similar communication signal evolved in humans, dogs, and cats. However, individual differences—specifically in personalities—exist. As dogs/cats and humans spend time together in a house, they can learn communications unique to the pair/group and probably form bonds with specific individuals. The bonds would be more beneficial for dogs and cats in terms of leading to more food, better food, and greater safety, and more beneficial for humans in terms of being less stressed, less anxious, and healthier. Therefore, interspecific bonding benefits both parties.

## Voices and Smell That May Contribute to Bond Formation

Since dogs and cats discriminate between humans using integrated different types of senses, other senses, such as auditory and olfactory, may also contribute to bond formation. Along with visual information, other forms of perceptual information may promote bond formation. Cats have adapted their voices to communicate more effectively with humans. For example, adult cats meow at humans (Mertens and Turner, 1988), but otherwise, meowing is generally only used for communication between kittens and their mothers (Bradshaw and Cameron-Beaumont, 2000). Additionally, domestic cats’ meows are more comforting to humans than those of wildcats (Nicastro, 2004), and feral cats’ meows are different from house cats’ in acoustic variables indicated by a spectrogram (Yeon et al., 2011). Another example is that cats purr more when they are reunited with their owners after a long separation (Eriksson et al., 2017). Purring is a general sign of contentment or care soliciting behavior (McComb et al., 2009). Although purring can occur in different contexts (Merola and Mills, 2016), it is most commonly seen in kittens to solicit care from mothers (Bradshaw and Cameron-Beaumont, 2000). It is considered that domestication and socialization have led to the development of a cat’s vocal communication with humans. Cats are also sensitive to human vocalizations. Cats distinguish between the voices of their owners and strangers (Saito and Shinozuka, 2013). Cats participating in a habituation-dishabituation test showed a decreasing response when strangers’ voices continued and increasing head and ear movements when hearing their owner’s voice. Dogs also distinguish the voices of their owners (Adachi et al., 2007) and read human emotions through voice and intonation (Andics et al., 2016).

Cats also have highly developed communication through smell. Most small felids, including *Felis silvestris lybica*, the ancestor of cats, have exclusive territories. Species with large territories rarely encounter others and tend to communicate through smell. Some cats live in multi-cat households and high-density urban environments. Cats living in groups may distinguish between individuals who are group members and non-group members through smell. Cross infection between individuals during allorubbing or while marking communal scent posts would increase any similarity of smell profiles among the members of a social group, but that is not conclusively demonstrated (Gittleman, 2013). Thus, communication through smell in cats may be more complex than in other felids. They also communicate with humans through smell, as they exhibit rubbing behavior. The connection between their rubbing and social bonding is supported by the fact that cats are adept at communicating through smell. Smell helps dogs distinguish between their owners and strangers. The caudate nucleus region of dogs’ brains is more strongly activated when exposed to familiar human smell compared to the smell of familiar dogs, unfamiliar dogs, unfamiliar humans, and their own smell, suggesting a positive emotional response to familiar human smell (Berns et al., 2015). Dogs may also distinguish human emotions by smell. Dogs showed higher cardiac activation when sniffing human fear chemosignals than when neutral (Siniscalchi et al., 2016). Dogs also show a similar emotional response to others through smell (D’Aniello et al., 2018) and behave accordingly.

Furthermore, dogs and cats generate visual images when they hear vocalizations (Adachi et al., 2007; Takagi et al., 2019). Such interchanges of information across sensory modalities may be useful to animals because the available modalities may be unavailable at other times. Additionally, individuals are identified through several senses, such as appearance, voice, and smell. The contribution of auditory and olfactory communication to bond formation will need to be investigated in future studies.

## Conclusion

Dogs and cats have both been integrated into human habits for improved access to food, and they use human signals to obtain information such as the location of food. However, they differ in their food acquisition situations. Dogs exhibit behaviors that require human cooperation while cats do not rely on humans, perhaps due to the nature of their ancestral species. Dogs first cooperated with humans as working animals, and cats were allowed in the human habitat to catch mice. However, today cats and dogs share an equal and similar ecological niche among humans.

It is particularly interesting that cats, originally solitary animals, can adapt to living in groups with humans and other cats. Most of the behaviors that cats exhibit toward humans were initially observed in mothers-kittens, suggesting that the behaviors seen in their adaptation to a group with humans were inherent. In the future, cats may acquire more dog-like abilities, such as more consistent and expressive gaze, through human selection. Investigating changes that cats may exhibit by selection would be helpful for understanding the evolutionary process of sociality in a broader context.

Dogs and cats use several senses to communicate with humans. Each of these senses contributes to the distinction between owner and stranger. Voices, smell, and other factors also foster the formation of bonds between dogs/cats and humans, and future research should investigate other perceptions that similarly may have been involved in the formation of bonds. The literature at present indicates that dogs and cats have their own adaptive communications that may have provided the basis for their mutually beneficial coexistence with humans.

## Author Contributions

HK, TK, ST, and MN wrote the manuscript. All authors contributed to the article and approved the submitted version.

## Conflict of Interest

The authors declare that the research was conducted in the absence of any commercial or financial relationships that could be construed as a potential conflict of interest.
