# Corrigendum: The Gaze Communications Between Dogs/Cats and Humans: Recent Research Review and Future Directions

**DOI:** 10.3389/fpsyg.2021.645366

**Published:** 2021-03-01

**Authors:** Hikari Koyasu, Takefumi Kikusui, Saho Takagi, Miho Nagasawa

**Affiliations:** ^1^Laboratory of Human-Animal Interaction and Reciprocity, Azabu University, Kanagawa, Japan; ^2^Japan Society for the Promotion of Science, Tokyo, Japan

**Keywords:** dogs, cats, humans, gaze, interaction, communication, bond

In the original article, there was a mistake in Table 1 as published. The references for the entry “Looked alternately at the food and the owner when it could access the food” (section “social reference”) were incorrect. The corrected Table 1 appears below.

**Table 1:** Gaze communication between dogs/cats and humans.

**Dogs**	**References**	**Cats**	**References**
**Response to human gaze**			
Stole food less often	Call et al., 2003; Kaminski et al., 2013	Avoided the gaze of familiar human	Koyasu and Nagasawa, 2019
Obeyed more commands of their owners	Schwab and Huber, 2006	Selected food from humans who looked at them	Ito et al., 2016
Fetched the toy that humans could see in the situation with two toys	Call et al., 2003		
Increased attention-getting behaviors	Ohkita et al., 2016		
Selected food from humans who looked at them	Gácsi et al., 2004		
**Using human signals**			
Used human pointing in the task of selecting one of two containers	Miklosi et al., 2005	Used human pointing in the task of selecting one of two containers	Miklosi et al., 2005
Used human gaze direction with pointing in the task of selecting one of two containers	Hare et al., 2002	Looked in the direction indicated by human gaze (with head movements)	Pongrácz et al., 2019
Looked in the direction directed by human gaze (with head movements)	Hare et al., 1998; Agnetta et al., 2000; Téglás et al., 2012; Met et al., 2014	Followed the container that humans visited in a situation with two food containers	Chijiiwa et al., 2020
Followed the container that humans visited in a situation with two food containers	Chijiiwa et al., 2020; Nagasawa et al., 2020		
**Social reference**			
Looked alternately at the food and the owner when it could access the food	Miklosi et al., 2005, Lazzaroni et al., 2020	Did not look alternately at the food and the owner when it could not access the food	Miklosi et al., 2005
Looked alternately at the strange object and the owner	Merola et al., 2012a,b	Looked alternately at the strange object and the owner	Merola et al., 2015
**The role of gaze in bond formation**			
Increased attention-getting behaviors in dogs, which function as attachment behaviors in response to human gaze	Ohkita et al., 2016	Eyeblink synchronization during mutual gazing	Koyasu et al., 2020
Dog owner's oxytocin secretion increased in response to the dog's gaze	Nagasawa et al., 2009		
An oxytocin-mediated positive loop of bond formation facilitated and modulated by gazing, like mother-infant	Nagasawa et al., 2015		
Eyeblink synchronization during mutual gazing	Koyasu et al., 2020		

The authors apologize for this error and state that this does not change the scientific conclusions of the article in any way. The original article has been updated.
